# Design, Delivery, Maintenance, and Outcomes of Peer-to-Peer Online Support Groups for People With Chronic Musculoskeletal Disorders: Systematic Review

**DOI:** 10.2196/15822

**Published:** 2020-04-24

**Authors:** Liam R Maclachlan, Kathryn Mills, Belinda J Lawford, Thorlene Egerton, Jenny Setchell, Leanne M Hall, Melanie L Plinsinga, Manuela Besomi, Pek Ling Teo, Jillian P Eyles, Rebecca Mellor, Luciano Melo, Sarah Robbins, Paul W Hodges, David J Hunter, Bill Vicenzino, Kim L Bennell

**Affiliations:** 1 The School of Health and Rehabilitation Sciences The University of Queensland Brisbane Australia; 2 Faculty of Medicine and Health Sciences Macquarie University Sydney Australia; 3 Centre for Health, Exercise & Sports Medicine Department of Physiotherapy The University of Melbourne Melbourne Australia; 4 Faculty of Medicine and Health, University of Sydney Kolling Institute of Medical Research Institute of Bone and Joint Research Sydney Australia; 5 Department of Rheumatology Royal North Shore Hospital Sydney Australia; 6 Sax Institute Sydney Australia

**Keywords:** social support, musculoskeletal diseases, online social networking, empowerment

## Abstract

**Background:**

Online support groups (OSGs) are one way for people with chronic diseases, their family or friends, and health professionals to communicate, gain information, and provide social support. As the number of peer-to-peer OSGs for chronic musculoskeletal conditions grows, it is important to gain insight into the different designs of groups available, who is accessing them, if and how they may be effective, and what strategies are being used to implement or increase consumer engagement.

**Objective:**

The objectives of this systematic review of people with musculoskeletal conditions were to (1) describe the design features (functions, usage options, moderation, and expert input) of peer-to-peer OSGs, (2) describe the characteristics of the individuals using peer-to-peer OSGs, (3) synthesize the evidence on outcomes of participation, and (4) identify strategies used in the delivery and maintenance of OSGs.

**Methods:**

A search comprising terms related to the population (people with musculoskeletal disorders) and the intervention (peer-to-peer OSGs) was conducted in 6 databases. Results were filtered from 1990 (internet inception) to February 2019. Studies identified in the search were screened according to predefined eligibility criteria using a 2-step process. Quantitative studies were appraised by 2 reviewers using the Risk Of Bias In Non-Randomized Studies of Interventions tool. Qualitative studies were appraised by 2 different reviewers using the Critical Appraisal Skills Programme checklist. Extracted data were synthesized narratively.

**Results:**

We examined 21 studies with low to moderate risk of bias. Of these studies, 13 studies included OSGs hosted on public platforms, 11 studies examined OSGs that were conducted in English, and 6 studies used moderators or peer leaders to facilitate engagement. Studies either reported the number of OSG members (n=1985 across all studies) or the number of posts (range: 223-200,000). The majority of OSG members were females who were not full-time employees and with varied levels of education. There were no randomized controlled trials measuring the efficacy of OSGs. Qualitative and quantitative studies identified empowerment, social support, self-management behavior, and health literacy as primary constructs to measure OSG efficacy. Neutral or marginal improvement was reported in these constructs. Sharing experiences and a greater level of engagement appeared to have an important influence on OSGs efficacy. The extent to which members posted on the website influenced engagement.

**Conclusions:**

Across a diverse range of designs, languages, included features, and delivery platforms, peer-to-peer OSGs for chronic musculoskeletal conditions attract predominantly female participants of all ages and education levels. The level of participation of a member appears to be related to their perceived benefit, health literacy, and empowerment. Future studies are needed to identify which design and maintenance strategies have superior efficacy and whether there are concomitant improvements in health outcomes for people with chronic musculoskeletal conditions resulting from participation in OSGs.

**Trial Registration:**

PROSPERO International Prospective Register of Systematic Reviews CRD42018090326; https://www.crd.york.ac.uk/prospero/display_record.php?ID=CRD42018090326

## Introduction

### Background

Chronic musculoskeletal disorders are highly prevalent [1], the leading cause of nonfatal disease burden [2], and include conditions (such as low back pain) that are the leading cause of disability internationally [3]. Musculoskeletal disorders disrupt daily living and account for a large proportion of lost productivity in the workplace [4]. Given that there is no cure for many chronic musculoskeletal disorders, long-term self-management is a core recommendation of clinical guidelines [5,6]. Central to effective long-term management is patient education and advice relating to medication, therapeutic exercise, general physical activity, weight loss (if appropriate), and potentially beneficial physical and psychological treatments [6-8].

Another key factor in the management of musculoskeletal disorders is social support, as it may positively influence health behaviors susceptible to social influence [9]. Social support may also buffer the negative impact of low health literacy [10]. Both are essential in negotiating health care systems [11] and may impact health outcomes. For example, in individuals with hip and knee osteoarthritis, increased social support has been associated with higher levels of health-related quality of life [12]. Conversely, in those with rheumatoid arthritis, low levels of social support at the time of diagnosis have been predictive of poorer functional disability and pain outcomes 5 years later [13].

Online support groups (OSGs) are one way in which people with chronic musculoskeletal disorders can access social support and information. OSGs range from self-initiated groups on social media (eg, Facebook) to custom-developed websites run by clinicians or organizations. Their common goal is to provide opportunities for people to share experiences, advice, and support for their chronic disorders [14]. Given that a United Nations report (December 7, 2018) reported that more than 50% of the world’s population now has access to the internet and that Web-based health service usage is increasing, OSGs may provide an accessible, convenient, and efficient means of augmenting social support and self-management. To date, the research pertaining to the characteristics of OSG platforms, group members, and implementation strategies is varied, and there is little focus on individuals with chronic musculoskeletal disorders. This makes it difficult to draw conclusions regarding if and how they are clinically effective or have a role in musculoskeletal health care.

### Objectives

This study aimed to systematically review the literature evaluating the use of peer-to-peer OSGs for people with chronic musculoskeletal disorders. The 4 objectives of this review were to (1) describe the design features of peer-to-peer OSGs, (2) describe the characteristics of individuals involved in peer-to-peer OSGs, (3) synthesize the evidence on the effectiveness of OSGs, and (4) identify implementation strategies used in the delivery of OSGs.

## Methods

### Review Registration

The Preferred Reporting Items for Systematic Reviews and Meta-Analyses statement was used to ensure complete reporting, and the review protocol was registered in the International Prospective Register of Systematic Reviews (CRD42018090326).

### Search Strategy

The search strategy was developed in consultation with a librarian from The University of Queensland and involved 2 components: the population (people with chronic musculoskeletal disorders) and the intervention (peer-to-peer online support). The full PubMed search strategy is shown in Multimedia Appendix 1. The following electronic databases were searched: PubMed, CINAHL, EMBASE, PsycINFO, Scopus, and PubMed Central. A time-based filter was implemented, capturing all potential studies from 1990 (year inception of the internet) to February 26, 2019. The search included both keywords and subject heading terms. Supplementary searches of reference lists of included studies were undertaken.

### Study Selection

Studies involving OSGs for adults (>18 years) with chronic (>3-month duration) musculoskeletal disorders (ie, disorder that primarily affects the musculoskeletal system) were considered eligible. Eligible interventions included any peer-to-peer (ie, participants interacting) OSG (>3 participants on an online platform) with or without moderation or expert input or supervision. Observational studies, cohort studies, case-control studies, randomized controlled trials, qualitative studies, and mixed method studies were eligible for inclusion.

Studies not available in English and studies of pediatric populations and animals were excluded. Telehealth interventions, where health care consultations are delivered remotely via phone or internet, were excluded. Studies that used online peer-to-peer support as part of a combined or complex intervention were only included if the OSG component of the intervention was examined as an independent component, and data were available for extraction. In studies that investigated a range of morbidities, extracted data were limited to those from individuals with musculoskeletal disorders. Studies in which data pertaining to musculoskeletal disorders were not presented separately and could not be extracted were included if musculoskeletal disorders accounted for the majority of cases and authors could provide these data when contacted. When multiple studies were identified from the same groups of authors, they were contacted to determine whether samples used were independent or the same across studies. When no response was received, samples that were similar in terms of musculoskeletal disorder and year of recruitment were assumed to be the same and included only once in the analysis.

Using the eligibility criteria described above, a 2-step process was used for screening and selection. Titles and abstracts of all identified studies were screened by any 2 of the 4 reviewers (LM, MP, MB, and RM) using Covidence (Covidence, Melbourne, Australia). Additional reviewers (KM, JE, and TE) were asked to resolve screening disagreements. Full-text articles of all eligible studies were retrieved and screened by any 2 of the reviewers mentioned above, with conflicts resolved by discussion.

### Data Extraction

The authors worked in 4 groups (1 for each research question) to extract data using custom-developed spreadsheets. For the first research question relating to the design features of OSGs, the following data were extracted: (1) presence and type of moderation or expert input; (2) functions and design features of host platforms; (3) content, frequency, and volume of member posts and information uploaded; and (4) involvement from participants. The second research question regarding member characteristics involved the extraction of demographics, roles and relationships, and health disorders. For the third research question relating to the effectiveness of OSGs, the following data were extracted: (1) the constructs by which effectiveness was measured, (2) outcome measures utilized to quantify effectiveness constructs, (3) processes and themes explaining any benefits, and (4) results of effectiveness studies or satisfaction ratings. For the fourth research question relating to implementation strategies, the following data were extracted: (1) group development and initiation strategies, (2) where the group was hosted, and (3) barriers and enablers to engagement in support groups.

### Assessment of Study Quality

Qualitative studies were evaluated with the Critical Appraisal Skills Programme (CASP) checklist [15]. The CASP involves 10 questions divided into 3 sections: (1) validity of the results (questions 1 to 6), (2) reporting of results (questions 7 to 9), and (3) utility of the results (question 10). Moreover, 2 of the 3 reviewers (JS, TE, and KM) independently assessed the included qualitative studies. Conflicts were resolved through discussion until consensus was reached.

Quantitative studies were appraised with the Risk Of Bias In Non-Randomized Studies of Interventions tool (ROBINS-I) [16]. The ROBINS-I assesses 7 domains of bias divided across 3 timepoints: preintervention (confounding and selection bias), at intervention (classification of the intervention), and postintervention (deviation from the intervention, missing data, measurement error, and reporting bias). A total of 2 authors (LH and KM) performed the assessment, with any conflicts resolved until consensus was reached. The overall risk of bias was determined by the triangulation of results across all domains.

### Data Synthesis

A narrative synthesis of findings was conducted because of the heterogeneity in the type of OSG, evaluation measures used, and population and designs of the included studies.

## Results

### Study Selection

**Figure 1:**
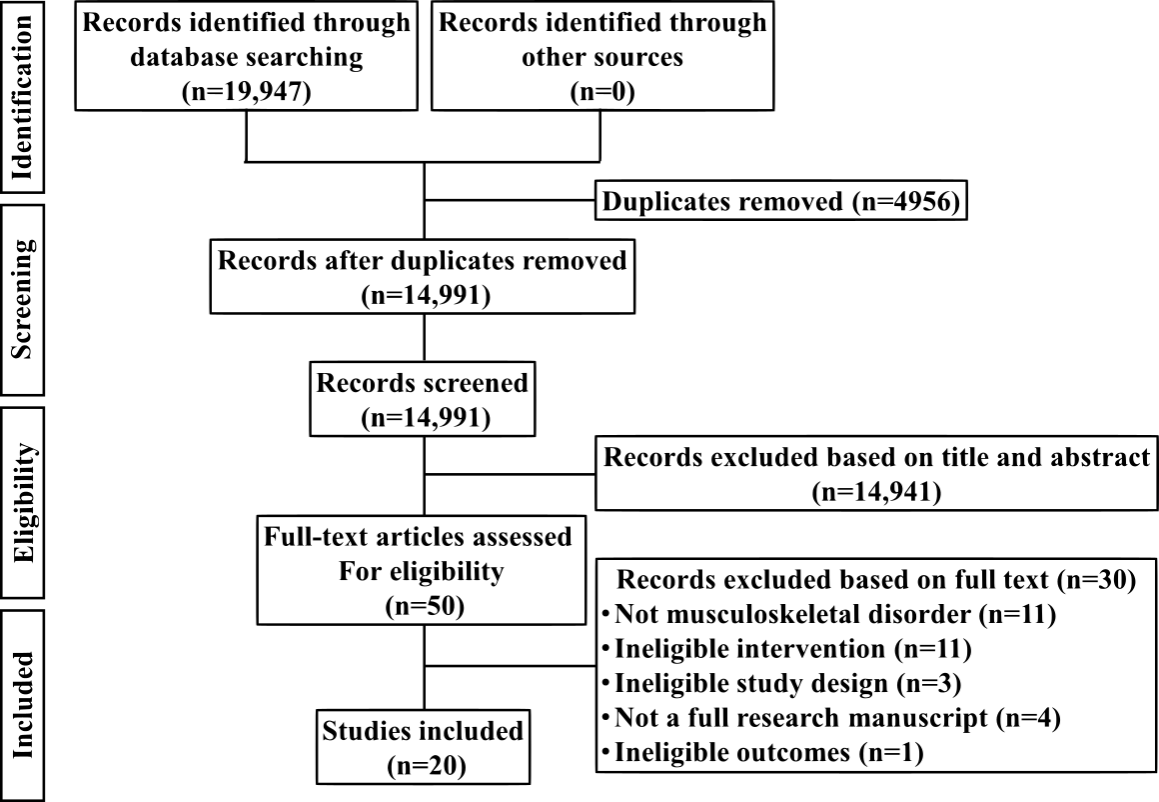
Study eligibility flow.

The process of study selection is shown in Figure 1. The search yielded 19,947 articles. Following the removal of duplicates, 14,991 titles and abstracts were screened. Of these, 50 full-text articles were considered, from which 20 studies were eligible for the review.

### Study Characteristics

Description of the design, sample size, and aims of the included studies is shown in Table 1. Overall, 10 studies were qualitative, 7 were quantitative, and 3 employed both qualitative and quantitative components. In terms of study design, 3 studies were prospective and the rest were cross-sectional. None of the studies were randomized controlled trials. We found 3 studies that used data from the same OSGs in the Netherlands but had different foci: forum leaders (n=32) [17], all participants (n=528) [18], or compared *posters* (people who write comments on OSG pages) with *lurkers* (people who read material without contributing posts; n=109) [19]. Moreover, 2 studies used the discourse of the same 20 members from 4 arthritis-related OSGs in the United States [20,21], and a third study by the same authors examined 1960 posts from the same 4 OSGs [22].

### Study Quality

#### Quality Assessment of Qualitative studies

Of the 20 included studies, 13 included a qualitative component. On average, studies met 7.4 (out of 10) CASP items. Most did not articulate how interviewer perspectives may have influenced their findings. One study met 3 of the 10 criteria, implying poor methodological quality and inability to confirm the validity of findings [23]. Multimedia Appendix 2 provides full details of the quality assessment of qualitative studies. A study [24] that described the design and development of an online community without undertaking a formal qualitative evaluation was excluded from the quality assessment.

**Table 1:** Design, sample size, and aims of included studies.

References	Country^a^	Study design	Sample size/sample volume	Study aim
Ammerlaan et al [25]	The Netherlands	Prospective feasibility (participant survey)	12 members	To test the feasibility of the Web-based and face-to-face self-management program
Bright et al [26]	United Kingdom	Retrospective online participant survey	152 respondents	To identify the characteristics and motivations of Web-based health information seekers accessing the Web-based health community
Camerini et al [27]	Switzerland	Retrospective online participant survey	209 respondents	To evaluate the effectiveness of an internet-based patient education intervention
Hadert and Rodham [28]	United Kingdom	Retrospective, qualitative, interpretive, phenomenological analysis	374 members, 1068 posts	To investigate how and why an arthritis Web-based message board was used
Shigaki et al [29]	United States	Retrospective, qualitative	30 participants	To evaluate social interactions among individuals with rheumatoid arthritis participating in an empirically based, cognitive-behavioral, self-management, peer support program delivered in a Web-based format
Smarr et al [24]	United States	Feasibility	114 members, 448 posts	To describe the Web-based transformation of an empirically validated, clinic-based, self-management program for rheumatoid arthritis
Smedley et al [30]	United Kingdom	Retrospective qualitative content analysis	23 members, 223 posts	To explore the experiences of members in a newly launched complex regional pain syndrome discussion forum to examine how support processes become established
Smedley et al [31]	United Kingdom	Retrospective, qualitative thematic analysis	59 moderators, 790 posts	To identify and describe the activities performed by Web-based support community moderators
van Uden-Kraan et al [17]	The Netherlands	Semistructured interviews	32 participants	To explore if, and in which ways, patients feel empowered by participation in OSGs^b^
van Uden-Kraan et al [32]	The Netherlands	Retrospective online participant survey	528 respondents	To explore if lurkers in Web-based patient support groups profit to the same extent as posters do
van Uden-Kraan et al [19]	The Netherlands	Retrospective qualitative content analysis	1500 posts	To explore the extent to which potential disadvantages actually occur when participating in OSGs
van Uden-Kraan et al [18]	The Netherlands	Retrospective online participant survey	528 respondents	To explore the extent to which patients feel empowered by their participation in OSGs and what processes occurring in these groups are related to the empowering outcomes
van Uden-Kraan et al [23]	The Netherlands	Semistructured interviews	23 Web-masters	To determine the success factors of OSGs for patients and the motives and goals of people who start such groups
van Uden-Kraan et al [33]	The Netherlands	Prospective participant survey	679 respondents	To explore factors that facilitate or impede engagement in face-to-face and Web-based peer support
van der Vaart et al [34]	The Netherlands	Prospective participant survey	227 respondents	To examine current disease-related internet use and intentions to use various Web-based support services on a hospital-based interactive health communication app of patients with rheumatic diseases
Walker [35]	United States	Retrospective qualitative content analysis	292 posts	To explore how a relatively new medium of a disease-specific Facebook group is used to address needs of people affected by thoracic outlet syndrome
Willis [21]	United States	Retrospective qualitative discourse analysis (ethnomethodology)	5 members, 8231 posts	To understand how patients with arthritis use Web-based health communities to exchange disease-related information to better manage their chronic disease
Willis [20]	United States	Retrospective qualitative discourse analysis (ethnomethodology)	8231 posts	To examine self-efficacy within the computer-mediated communication of 4 Web-based health communities used by people with arthritis
Willis and Royne [22]	United States	Retrospective quantitative content analysis	1960 posts	To examine the computer-mediated communication within Web-based health communities for evidence of chronic disease self-management behaviors
Xing et al [36]	United States	Retrospective content analysis and survival analysis	100,000 users, 200,000 user posts	To understand how requests for and provisions of informational support by members with different social roles influence members’ continued participation in Web-based health communities

^a^Origin of online support groups when they are multinational.

^b^OSG: online support group.

#### Quality Assessment of Quantitative Studies

Of the 20 included studies, 10 included a quantitative component (Multimedia Appendix 3). Overall, 7 studies were rated as low risk of bias [22,27-33], and 3 studies were rated as moderate risk of bias [18,29]. Of the studies with a moderate risk of bias, 2 used multiple outcome measures to describe or quantify a single variable or concept, subsequently performing multiple analyses on a single research question [18,19].

### Design Features of Online Support Groups

Characteristics of the design and features of the OSGs are described in Tables 1 and 2. Of the 20 studies, 13 (65%) used platforms that were publicly accessible, 7 (36%) [24,26,34] were private platforms designed specifically for the study, and 1 study (15%) did not report the type of platform [33]. The most common type of platform was a purpose-built website (13/20, 65%). English language platforms were used by 52% (11/20) studies [20-22,24,26,28-31,35,36], whereas 42% (8/20) studies used Dutch platforms [17-19,23,25,32-34] and 1 study used an Italian platform [27]. There were 6 studies [21,24,25,28,30,31] that reported the number of OSG members, which ranged from 12 to 374 people (Table 1). The number of posts examined for content was reported by 10 studies [20-22,24,28,30-32,35,36], ranging from 223 to 200,000 (Table 1). The average duration of membership for the platforms ranged from 4 weeks to 6 years.

Moderation of the OSG was used in 6 studies [23-25,30-32] (Table 2). Moderators were participants with musculoskeletal disorders (4 studies) [23,25,31,32], health professionals (1 study) [24], and organizers or administrators (1 study) [30]. The type of moderation consisted of supportive tasks, sharing experiences, facilitating information sharing, making announcements, administrative tasks (eg, removal of *disadvantaged* posts, monitoring members’ activity, and maintaining the rules of the OSG), and leading group activities (eg, chat and discussion forums).

**Table 2:** Description of online support groups included for review.

References	Target population	Type of platform	Duration of OSG^a^	Language	Frequency of posts	Presence/source of moderation
Ammerlaan et al [25]	Young adults (age 16-25 years) with arthritis	Private website; planned weekly chat group (90 min)	6 weeks	Dutch	NR^b^	Yes/peer
Bright et al [26]	Adults with knee problems	Private website *KNEEguru*	1 month	English	NR	No
Camerini et al [27]	Adults with FMS^c^	Private website; also included video and textual material on coping	Mean 167 days (SD 67.6)	Italian	NR	No
Hadert and Rodham [28]	Adults with arthritis	Public website	3 months	English	NR	No
Shigaki et al [29]	Adults with RA^d^	Private website	10 weeks	English	NR	No
Smarr et al [24]	Adults with RA	Private website with multiple shared resources (eg, education material and audio files)	Average of 10 weeks	English	NR	Yes/health professional
Smedley et al [30]	Adults with CRPS^e^	4 private forums	6 months	English	17=low frequency posters^f^ (average 9.5 posts); 6=high-frequency posters	Yes/peer
Smedley et al [31]	Adults with arthritis, CRPS, Crohn disease, depression, Huntington disease, and diabetes	6 public discussion forums	NR	English	15 posts per moderator	Yes/peer
van Uden-Kraan et al [17]	Adults with arthritis, FMS, or breast cancer	9 public websites	NR	Dutch	Posters >1/day=140; 1/day=121; >1/week=96; 1/week=31; 1/month=6; and <1/month=6	No
van Uden-Kraan et al [32]	Adults with arthritis, FMS, or breast cancer	8 public websites	1 year (range 0-6 years)	Dutch	Minimum=1/day	Yes/peer
van Uden-Kraan et al [19]	Adults with arthritis, FMS, or breast cancer	8 public websites	3 months	Dutch	1 or 2 messages	No
van Uden-Kraan et al [18]	Adults with arthritis, FMS, or breast cancer	Public websites	Up to 2.5 years	Dutch	Posters >1/day=146; 1/day=139; >1/week=124; 1/week=50; 1/month=13; and <1/month=13	No
van Uden-Kraan et al [23]	Adults with arthritis, FMS, or breast cancer	10 Public websites, 13 private websites, 18 stand-alone (not embedded in organization website/forum) OSGs, and 5 patient advocacy websites	NR	Dutch	Ranged from a few messages per week to hundreds of messages daily	Yes
van der Vaart et al [34]	Individuals with rheumatic diagnosis	Private app	NR	Dutch	NR	No
Walker [35]	Adults with thoracic outlet syndrome	Public; hosted on Facebook	7 months	English	NR	NR
Willis [21]	Adults with arthritis	4 public websites	NR	English	Once every 4 days	No
Willis [20]	Adults with arthritis	4 public websites	NR	English	Only high-frequency posters participated	No
Willis and Royne [22]	Adults with arthritis	4 public websites	4 weeks	English	NR	No
Xing et al [36]	Individuals with (or associated with) FMS	Public website	Up to 6 years	English	Core group members: average of 393 posts; peripheral members: 9.58 posts	Possible

^a^OSG: online support group.

^b^NR: not reported.

^c^FMS: fibromyalgia.

^d^RA: rheumatoid arthritis.

^e^CRPS: complex regional pain syndrome.

^f^Poster: people who write comments on online support group pages.

### Characteristics of Individuals Involved in Online Support Groups

Participant characteristics were reported to varying degrees of detail across studies (Table 3). Participants’ age was reported in 8 studies [20,25-27,29,30,33,34], which ranged from 18 to 83 years. Of the 1370 participants in the 8 studies reporting gender [20,25-27,29,30,33,34], 1092 (80%) were female. Education history was reported in 6 studies [25-27,29,33,34]. Of the 1252 participants accounted for, 499 (39%) had a maximum of *low-tier education*, but this category was not defined by the studies’ authors. Occupational status was reported in 3 studies [26,33], accounting for 1068 participants, of which 643 (60%) were unemployed. Relationship status was reported in 4 studies [26,29,33,34], with 836 of 1071 (78%) participants being married or cohabiting. All studies stated their disorder of interest. The most commonly encountered musculoskeletal disorders were unspecified types of arthritis [17-23,25,28,32,33] and fibromyalgia [17-19,23,25,27,32,36]. Moreover, 5 studies examined individuals with rheumatoid arthritis [24,25,29,33,34], and 2 studies each investigated rheumatic disease [28,34], chronic regional pain syndrome [30,31], and spondyloarthropathy [25,28].

Not all participants in each of the OSGs had a musculoskeletal problem (Table 3). Of the 15 studies that identified the roles of OSG members, 2 studies identified that health professionals were included in the group [24,32], and 3 studies included family members or acquaintances of people with the disorder [23,32,35]. Within groups of patient members, 3 studies identified that members could either be participants (n=292) or moderators or peer leaders (n=66) [25,31,32], and 2 studies separated members into active posters (core members) (n=460) or lurkers (peripheral members) (n=9429) [19,36]. *Peripheral members* were noted to post significantly less frequently (mean 9.9 posts, SD 21.6) than *core members* (mean 393.5 posts, SD 372.9) [36], and *lurkers*, as by definition, did not post at all [19].

**Table 3:** Characteristics of online support group users.

References	Age (years), mean (range or SD)	Gender (female/male)	Education levels	Occupation	Marital status	Motivation for joining
Ammerlaan et al [25]	22 (range: 17-25)	9/1	Vocational training: 1; advanced vocational training: 7; college/university: 2	N/A^a^	N/A	N/A
Bright et al [26]	40.1	93/59	Higher education qualifications: 114	Employed: 87; unemployed: 65	Cohabiting: 104	Emotional support (clarity regarding advice and treatments), social support (sharing experiences and information), and condition support (achieving a sense of authority)
Camerini et al [27]	49 (range: 25-74)	199/10	8 years of schooling: 36; high school/university: 163; not reported: 10	N/A	N/A	N/A
Hadert and Rodham [28]	N/A	N/A	N/A	N/A	N/A	Needing to be believed, information exchange, sharing support, and sharing emotions
Shigaki et al [36]	49.4 (range: 30.1-68.5)	28/2	Mean years of education: 15 (range: 12-20) years	N/A	Married: 19	N/A
Smarr et al [24]	N/A	N/A	N/A	N/A	N/A	N/A
Smedley et al [30]	36.6 (range: 20-54)^b^	18/5	N/A	N/A	N/A	N/A
Smedley et al [31]	N/A	N/A	N/A	N/A	N/A	N/A
van Uden-Kraan et al [17]	43 (range: 21-75)	30/2	Lower: 5; medium: 14; high: 13	Unemployed/unable to work: 25; employed: 7	Married/cohabiting: 26; not married: 6	N/A
van Uden-Kraan et al [32]	Posters^c^: 43 (SD 10.4); lurkers^d^: 47 (SD 9.9)	Posters: 392/27; lurkers: 102/7	Posters—lower: 129; medium: 170; high: 111. Lurkers—lower: 42; medium: 43; high: 24	Posters—working >20 hours: 128; working ≤20 hours: 54; unemployed: 234. Lurkers—working >20 hours: 39; working ≤20 hours: 11; unemployed:59	Posters—in a relationship: 331; single: 88. Lurkers—in a relationship: 85; single: 25	N/A
van Uden-Kraan et al [19]	38 (range: 21-65)	293/29; unknown: 25	N/A	N/A	N/A	N/A
van Uden-Kraan et al [18]	44 (range: 17-75)	494/34	Lower: 171; medium: 213; high: 135	Working >20 hours: 167; working ≤20 hours: 65; unemployed: 293	In a relationship: 415; single: 113	N/A
van Uden-Kraan et al [23]	46 (range: 24-65)	20/3	N/A	N/A	N/A	Provide information and social support
van Uden-Kraan et al [33]	54 (range: 18-75)	571/106	Lower: 404; medium: 176; high: 94	Employed: 212; unemployed: 447	Married/cohabiting: 530; single: 128	Improve mental health and past behaviors with support groups
van der Vaart et al [35]	52 (SD 11)	143/84	Lower: 61; average: 116; high: 46; unknown: 4	Employed: 119; unemployed: 106	Married/cohabiting: 183; single: 42; unknown: 2	Poor mental health and improving health literacy^e^
Walker [35]	N/A	N/A	N/A	N/A	N/A	N/A
Willis [21]	Range: 21-83	15/5	N/A	N/A	N/A	N/A
Willis [20]	Range: 21-83	15/5	N/A	N/A	N/A	N/A
Willis and Royne [22]	N/A	N/A	N/A	N/A	N/A	N/A
Xing et al [36]	N/A	N/A	N/A	N/A	N/A	N/A

^a^N/A: not applicable.

^b^Age was available for 9 participants, and the duration of symptoms was available for 14 participants.

^c^Poster: people who write comments on online support group pages.

^d^Lurker: people who read material without contributing posts to the forum.

^e^People with good health literacy were more likely to use peer support services to further improve knowledge.

### Effectiveness of Online Support Groups

Overall, 10 studies reported on measures of effectiveness from OSGs [17-22,25,27,29,30]. Effectiveness was conceptualized as the development of patient empowerment [17-19], social support [25,29,30], self-management processes [20,22,27], and health literacy [21,27]. In evaluating effectiveness, none of the studies considered clinical domains (eg, pain or physical function; Multimedia Appendix 4).

Themes and processes of developing social activity, empowerment, self-management, and health literacy were explored by 4 studies using qualitative study designs [17,20,29], by 5 studies using quantitative designs [18,19,22,27,30], and by 1 study using mixed method [25]. Using semistructured interviews with 32 OSG users with arthritis, fibromyalgia, or breast cancer, van Uden-Kraan et al [17] concluded that patient empowerment was achieved by (1) being better informed, (2) feeling more confident, (3) increasing social well-being and enhanced self-esteem, and (4) acceptance and coping with chronic disease. Information and support were also found to be important themes for developing self-management and social support. Moreover, 2 studies reported that approximately one-third of all user posts contained these themes [21,30]. Sharing personal and disease experiences, particularly from disease veterans, was important in the development of a social activity, self-management plans, and improving health literacy [20,25]. This frequently included posts on drug management (29.3%) and symptom management (22.7%) [22]. Other common themes in these latter 3 effectiveness domains were seeking emotional support, positive feedback, and reinforcement from the community [20,21,25,30] (Multimedia Appendix 4).

When quantifying the effectiveness of OSG participation, participants with arthritis aged 25 years or younger reported high levels (mean 8.4, range: 6-10) of satisfaction with goal attainment, using a 10-point numerical rating scale [25]. However, a survey of 528 individuals with fibromyalgia, arthritis, and breast cancer indicated that they were neutral or in agreement (scores of 3-4 on a 5-point Likert scale) with their achievement of (1) being better informed, (2) enhancing social well-being, and (3) improving illness acceptance as a result of their participation in an OSG [18]. Further exploration of these findings revealed that people who were more engaged, evidenced by visiting the site more frequently or making more posts, experienced greater gains in health literacy, self-esteem, and self-management than those who made fewer posts or lurked [19,27] (Multimedia Appendix 4). Willis and Royne [22] reported that improvements in mobility, flexibility, pain, and energy were among the most frequently reported benefits of participation across 4 arthritis OSGs; however, they also reported significant differences between OSGs, suggesting that the perceived benefits may be specific to a group. Multimedia Appendix 4 summarizes all measures used to investigate the effectiveness of OSGs.

### Implementation Strategies to Deliver Online Support Groups

Overall, 7 studies [22,36] reported on the strategies used to implement OSGs. Groups tended to be either self-initiated by an individual sufferer of the disorder or by official consumer associations [32]. The latter were embedded within pre-existing websites containing health information, being either open access or available to subscribed members only [23]. We found 2 studies that used relevant stakeholders such as program moderators and/or patients in the development and testing of their OSG [24,25] and for its delivery [25].

One study reported that a key component of OSGs was to continually promote the group and keep it alive, which took considerable time and energy [23]. Strategies to do this included moderation, augmented learning, or a small core group of individuals who posted more frequently than more peripheral users. Moderating a group took approximately 10 to 15 hours per week (unspecific group size), which was often perceived by individual moderators as onerous [23]. In addition to moderation, 2 OSGs augmented learning by scheduling weekly group chats or setting homework tasks that centered around predetermined themes [24,25].

Member engagement, or staying in the OSG, was significantly associated with starting or contributing to threads and requesting information. Xing et al [36] reported that OSG members who start or contribute to threads 1 SD more frequently than average (range: 222 posts to 373 posts depending on group roles) were 20% more likely to stay engaged with the community. Similarly, OSG members who requested information 1 SD more frequently than average (approximately 16 information requests) were 29.3% more likely to remain in the group. Responding to questions or information requests also influenced member engagement. Posted questions generally received an answer within 24 hours, though a small number (15%) of questions received no answer at all [37]. If an information request was responded to by someone other than a *core group* member (ie, a peripheral group member), the person who made the request was 11.4% more likely to leave the group [36]. Criticisms of OSG implementation were that the discussion posts contained casual chitchat [32], and some OSGs had become *social clubs* rather than a place to exchange information and share experience [23].

## Discussion

### Principal Findings

This systematic review has revealed that the design features and implementation strategies used by peer-to-peer OSGs for people with chronic musculoskeletal disorders vary widely. People across a broad demographic spectrum access OSGs; some people chose to post actively, whereas others take a passive approach. Self-efficacy, health literacy, and empowerment are the constructs most commonly explored in studies investigating the effectiveness of musculoskeletal-focused OSGs. Overall, the findings stimulate discussion around optimal design and implementation of OSGs as well as how their effectiveness might best be measured. These topics are recommended for future investigation, particularly for people with chronic musculoskeletal disorders.

### Comparison With Prior Work

For individuals with chronic musculoskeletal disorders, accessibility to OSGs is not influenced by whether the group is publicly or privately hosted. On the basis of the available literature, this also seems to be the case for OSGs focused on individuals with opioid addiction [38], depression [37], and asthma [39]. The majority of OSGs included in this review were hosted on public platforms. Previously, issues regarding privacy and security offered to users of public platforms have been raised [40]. A study of Facebook users comparing the amount and type of information disclosed on public and private Facebook groups indicates that private groups may be preferred, especially by people with social anxiety, because of the perception of greater control over who people are communicating with as well as greater trust and security of their information [41]. Our findings suggest that privacy and security were not barriers to participation in OSGs for people with chronic musculoskeletal disorders, and they did not influence the themes of information being shared. This may, however, have been because of the majority of group members also having the focus disorder, rather than the wider social network found on Facebook; the prevalence of the disorders within the general community; or the similarity in characteristics between group members (ie, primarily females who were not currently working). It appears that for people with chronic musculoskeletal disorders, the internet provides acceptable accessible sources of peer support for individuals seeking it, regardless of the hosting platform.

When examining the characteristics of OSG members included in this review, the majority of musculoskeletal-focused OSG members were female, not currently in full-time employment, and cohabitating or married. There is a significant association between exhibiting a preference for Web-based communication and the duration of internet usage [42]. Web-based communication is one way for people who are not working full time to maintain social activity when their peers and partners are not present. An explanation for the higher proportion of females in OSGs could be that although men use the internet more, women have been faster to adopt and are more frequent users of social networking and Web-based chat programs [43]. Furthermore, arthritis and fibromyalgia, the musculoskeletal disorders most commonly encountered in this review, are more common in females [44,45]. Another factor explaining lower male representation may be the perceived stigmatization of men sharing disease experiences on the Web [46]. Increasing representation of men in OSGs may be one way to improve self-management of disorders such as low back pain, the leading cause of years lived with disability for males since 1990 [2].

In evaluating OSG effectiveness, this review found that studies focused on constructs such as empowerment, self-efficacy, confidence, social support, and knowledge. These outcomes are consistent with those reported across multiple OSGs [47]. However, the lack of randomized controlled trials means that no causal inference can be established regarding OSG participation and change in these constructs. Self-efficacy has been identified as a foundation of chronic disease self-management [48], and multiple cohorts and observational studies of nonmusculoskeletal disorders have reported significant positive effects on self-efficacy following participation in OSGs and peer mentoring [40,47,49,50]. Findings from this review suggest that the extent to which OSG participation results in individuals with chronic musculoskeletal disorders feeling informed, confident, accepting of their disease is limited [18,19]. Furthermore, individuals who lurked, or did not actively post to OSGs, scored lower in the constructs of social well-being and self-esteem than active posters [19]. Although the direction of this relationship cannot be determined (active participation in OSGs leading to higher levels of social well-being and self-esteem, or vice versa), these results suggest that the type of participation may have a mediating effect. This has important implications for the implementation of future OSGs, as it appears that efforts must be made to engage individuals actively to contribute to posts, share stories, or ask questions [40].

One potential implementation method to promote active posting among OSG participants is the presence of a professional moderator [51]. Less than one-third of the studies included in this review reported the presence of a moderator. Of these moderators, the majority were peers who had the focus disorder. Although there does not appear to be a difference in OSG effectiveness irrespective of whether the moderator is a peer or health professional [40,51], Young et al [38] observed high attrition rates among peer moderators themselves. The time burden and onerous tasks involved in peer moderation may be one reason for this. Furthermore, when OSG member queries are not responded by peer moderators or leaders, general group attrition increased [36]. A previous review of OSGs [47] identified that attrition rates are lower with professional moderators. As such, having health professionals as moderators may be one way to address attrition rates and engagement. Health professional involvement may also help alleviate some of the time burden associated with moderating and administration for the group.

Additional implementation strategies that were investigated by studies included in this review were pretesting of OSGs before wider release, embedding the OSG in familiar websites, and scheduling weekly events or homework. No study investigated or reported the effectiveness of these strategies. Having identified these implementation strategies, a recommended topic for future research would be comparing the success of such implementation strategies with respect to consumer engagement and efficacy.

### Limitations

There are limitations that need to be considered when interpreting the findings of this review. The main limitation is that the health disorders of interest in several included studies were diverse, and in some cases, it was not possible to identify which data came from individuals with musculoskeletal disorders. There were 6 studies [17-19,23,31,32], primarily from a single research group, where data from individuals with musculoskeletal disorders could not always be distinguished from those with other chronic disorders. People with musculoskeletal disorders account for the majority of participants included in our data synthesis. Second, several included studies reported on the same group of OSGs. Although each of these studies explored different aspects of OSGs, the smaller overall sample limits generalizability. Third, many of the studies investigating the content of OSG posts only reported a summary of the most frequently occurring topics. As the general posting rate was low, this would overrepresent the attitudes and beliefs of individuals who were more actively engaged with the group. Fourth, all studies also focused on individuals who were already members of OSGs and often collected cross-sectional data, so it is impossible to determine change or development in outcomes over time. As such, it is difficult to attribute attitudes and beliefs regarding empowerment and self-efficacy to participation in OSGs or whether these were views formed before participating. Finally, as the focus of effectiveness evaluation was on attitudes and beliefs rather than health outcomes, the impact that OSGs have on clinical features and symptoms of musculoskeletal disorders could not be evaluated.

### Conclusions

OSGs provide an opportunity for individuals with musculoskeletal disorders to support one another through the sharing of knowledge and experiences. Across the diverse range of designs, languages, included features, and delivery platforms, OSGs attract participation from people of all ages and education levels, although predominantly females. The level to which group members participate appears to be related to their perceived benefit in health literacy and empowerment. However, the lack of control groups in studies means that direct inferences cannot be assessed or established. Participation may be increased by strategies such as moderation or input by a health professional or expert peers, homework tasks, and scheduled weekly chats. Whether these strategies are effective requires further investigation.

## Appendix

Multimedia Appendix 1 Search strategy built and conducted in PubMed.

Multimedia Appendix 2 Results of the quality assessment of qualitative study methods using the Critical Appraisal Skills Programme criteria.

Multimedia Appendix 3 Risk Of Bias In Non-Randomized Studies of Interventions for quantitative design studies.

Multimedia Appendix 4 Themes and magnitude of measure used to investigate the effectiveness of online support groups.

## Abbreviations

CASP: Critical Appraisal Skills Programme
NHMRC: National Health and Medical Research Council
OSG: online support group
ROBINS-I: Risk Of Bias In Non-Randomized Studies of Interventions
