# Supplementary material for: Design, Delivery, Maintenance, and Outcomes of Peer-to-Peer Online Support Groups for People With Chronic Musculoskeletal Disorders: Systematic Review
Source: J Med Internet Res. 2020 Apr 24;22(4):e15822. doi: 10.2196/15822 (PMC7210497; doi:10.2196/15822)
Supplement: Multimedia Appendix 1 [file jmir_v22i4e15822_app1.docx]

Appendix 1: **Search strategy built and conducted in PubMed**

(((((((((((((((((((((((((("Online support") OR "web support") OR "E* support") OR "chat room") OR "chat group") OR "newsgroup") OR "virtual support") OR "Electronic group") OR "Applications") OR "Mobile Applications"[MeSH]) OR "message board") OR "Peer Influence"[MeSH]) OR "Peer Influence") OR "peer to peer") OR "peer support") OR "social network") OR "Bulletin board") OR "Social Media"[MeSH]) OR "Social Media") OR "Twitter") OR "Tweet*") OR "Facebook"))))) AND ((((((((((((((((((((((((((((((((((("Musculoskeletal Pain"[Mesh]) OR "musculoskeletal pain")) OR "Musculoskeletal Diseases"[Mesh])) OR "Musculoskeletal Diseases")) OR "Musculoskeletal")) OR "Musculoskeletal condition*")) OR "Fibromyalgia"[Mesh])) OR "Fibromyalgia")) OR "Fatigue Syndrome, Chronic"[Mesh])) OR "Fatigue Syndrome, Chronic")) OR "complex regional pain syndrome")) OR osteoarthritis)) OR "Osteoarthritis"[Mesh])) OR arthritis)) OR "Joint Diseases"[Mesh])) OR "Joint Diseases")) OR "low back pain")) OR "Low Back Pain"[Mesh ]))
