# Supplementary material for: Design, Delivery, Maintenance, and Outcomes of Peer-to-Peer Online Support Groups for People With Chronic Musculoskeletal Disorders: Systematic Review
Source: J Med Internet Res. 2020 Apr 24;22(4):e15822. doi: 10.2196/15822 (PMC7210497; doi:10.2196/15822)
Supplement: Multimedia Appendix 2 [file jmir_v22i4e15822_app2.docx]

Appendix 2: Results of the qualitative study methods quality assessment using the CASP criteria

| Author | 1. Clear statement of the aims | 2. Qualitative methodology appropriate | 3. Research design appropriate | 4. Recruitment strategy appropriate | 5. Data collected to address aims | 6. Relationships considered | 7. Ethical issues considered | 8. Sufficiently rigorous data analysis | 9. Clear statement of findings | 10. Includes data related to review question(s) |
| --- | --- | --- | --- | --- | --- | --- | --- | --- | --- | --- |
| Ammerlaan, et al., [33] | No | + | No | + | No | No | + | No | No | + |
| Bright, et al., [34] | + | + | No | + | No | + | + | No | + | + |
| Hadert& Rodham [35] | + | + | + | + | + | + | + | + | + | + |
| Shigaki et al., [30] | + | + | + | + | + | No | + | No | +. | + |
| Smedley et al., [26] | + | + | + | + | + | No | + | + | + | + |
| Smedley et al 2017 [32] | + | + | + | + | + | No | + | + | + | + |
| van Uden-Kraan, et al., [17] | + | + | + | + | + | No | No | + | + | + |
| van Uden-Kraan, et al., [36] | + | + | + | + | + | No | No | + | + | + |
| van Uden-Kraan, et al. [19] | + | + | + | + | + | No | No | + | + | + |
| van Uden-Kraan, et al., [23] | + | + | No | No | No | No | No | No | No | + |
| Walker [27] | + | + | No | + | + | No | No | No | No | + |
| Willis [21] | + | + | + | + | + | No | + | + | + | + |
| Willis [20] | + | + | + | + | + | No | + | + | + | + |
