# Supplementary material for: Design, Delivery, Maintenance, and Outcomes of Peer-to-Peer Online Support Groups for People With Chronic Musculoskeletal Disorders: Systematic Review
Source: J Med Internet Res. 2020 Apr 24;22(4):e15822. doi: 10.2196/15822 (PMC7210497; doi:10.2196/15822)
Supplement: Multimedia Appendix 3 [file jmir_v22i4e15822_app3.docx]

Appendix 3: ROBINS-I for Quantitative design studies

|  | **Pre-intervention** | | | | | | | | **Intervention Period** | | | | **Post-Intervention** | | | | | | | | | | | | | | | | | | | | | | | **Overall risk** |  |  |
| --- | --- | --- | --- | --- | --- | --- | --- | --- | --- | --- | --- | --- | --- | --- | --- | --- | --- | --- | --- | --- | --- | --- | --- | --- | --- | --- | --- | --- | --- | --- | --- | --- | --- | --- | --- | --- | --- | --- |
| Risk of bias due to | Confounding | | | Selection of participants | | | | | Classification of interventions | | | | Deviations from intended intervention | | | | | | Missing data | | | | | | | Measurement of outcomes | | | | | | Reported results. | | | |  | | |
| Question | 1.1 | Risk | 2.1 | | 2.2 | 2.3 | 2.4 | Risk | 3.1 | 3.2 | 3.3 | Risk | 4.3 | 4.4 | 4.5 | 4.6 | Risk | 5.1 | | 5.2 | 5.3 | 5.4 | 5.5 | Risk | 6.1 | | 6.2 | 6.3 | 6.4 | Risk | 7.1 | | 7.2 | 7.3 | Risk |  | |  |
| Camerini, et al., [30] | No | Low | No | | No | NA | Yes | Low | Yes | Yes | No | Low | NI | NI | Partial Yes | NA | Low | NI | | NI | NI | NA | NI | Mod | No | | No | Yes | No | Low | No | | No | Partial Yes | Low | Low | |  |
| Shigaki, et al., [31] | No | Low | Partial Yes | | Partial No | NI | Yes | Mod | Yes | NI | No | Low | NI | NI | NI | NA | NI | Yes | | No | No | NA | NA | Low | NI | | NI | Yes | NI | Low | Partial No | | Partial Yes | No | Mod | Mod | |  |
| Smedly et al., [32] | No | Low | No | | NA | NA | Yes | Low | Yes | NI | No | Low | NI | NI | NI | NA | NI | Yes | | No | No | NA | NA | Low | NI | | NI | Yes | NI | Low | No | | No | No | Low | Low | |  |
| Van der Vaart, et al., [28] | No | Low | No | | NA | NA | Partial Yes | Low | Yes | Yes | No | Low | NI | NI | NI | NA | NI | Partial No | | Yes | Yes | NA | No | Mod | NI | | NI | Yes | NI | Low | No | | No | No | Low | Low | |  |
| Van Uden-Kraan, et al., [25] | No | Low | No | | NA | NA | Yes | Low | Yes | Yes | No | Low | NI | NI | NI | NA | NI | Partial No | | No | NI | NI | NA | Low | NI | | NI | Yes | NI | Low | No | | No | No | Low | Low | |  |
| Van Uden-Kraan, et al., [36] | No | Low | No | | NA | NA | Yes | Low | Yes | NI | No | Low | NI | NI | NI | NA | NI | Yes | | No | No | NA | NA | Low | NI | | NI | Yes | NI | Low | Partial Yes | | Partial Yes | Partial Yes | Mod | Mod | |  |
| van Uden-Kraan, et al., [18] | No | Low | No | | NA | NA | Yes | Low | Yes | NI | No | Low | NI | NI | NI | NA | NI | Yes | | No | No | NA | NA | Low | NI | | NI | Yes | NI | Low | Partial Yes | | Partial Yes | Partial Yes | Mod | Mod | |  |
| Walker., [25] | No | Low | Partial No | | NA | NA | Yes | Low | Yes | NI | NI | NI | NI | NI | NI | NA | NI | NI | | NI | NI | NA | NA | NI | NI | | NI | NI | NI | NI | No | | No | No | Low | Low | |  |
| Willis & Royne [22] | No | Low | No | | NA | NA | Yes | Low | Yes | NI | No | Low | NI | Yes | NI | NI | NI | Yes | | No | No | NA | NA | Low | No | | Yes | Yes | No | Mod | No | | No | No | Low | Low | |  |
| Xing, et al., [29] | No | Low | No | | NA | NA | No | Low | Yes | Yes | No | Low | NI | NI | NI | NA | NI | Yes | | No | No | NA | NA | Low | No | | Yes | Yes | No | Mod | No | | No | No | Low | Low | |  |

Scores are made as No/Partial No/Partial Yes/Yes or No Information (NI). Mod = moderate. NA = Non-applicable as tool indicates this item is skipped based on previous responses
