# Supplementary material for: Design, Delivery, Maintenance, and Outcomes of Peer-to-Peer Online Support Groups for People With Chronic Musculoskeletal Disorders: Systematic Review
Source: J Med Internet Res. 2020 Apr 24;22(4):e15822. doi: 10.2196/15822 (PMC7210497; doi:10.2196/15822)
Supplement: Multimedia Appendix 4 [file jmir_v22i4e15822_app4.docx]

**Table 4.** Themes and magnitude of measure used to investigate the effectiveness of online support groups.

| References | Type of efficacy | Identified theme (qualitative design) | Outcome measure utilized (quantitative design) | Quantitative finding |
| --- | --- | --- | --- | --- |
| Ammerlaan et al [33] | Social activity descriptions | Sharing experiences, strategies for self-management, social support, and feedback on goals and goal setting | Goal attainment scale (0-10) | Average 8.4/10 (range 6-10) |
| Camerini et al [30] | Health literacy and self-management processes | — | Literacy: 10 multiple-choice questions (symptoms and prevalence on fibromyalgia, treatment options, medications, and self-management strategies); self-management: amount of physical activity (5-point scale) | Health literacy of patients using the frequent asked questions section up to 6 to 10 times was significantly higher than those using the section up to 5 times  Self-management: 1 score increase in health literacy increased physical activity by 0.06 units |
| Shigaki et al [31] | Social activity descriptions | Appreciation of perceived support using interpersonal qualities (eg, friendliness and helpfulness), appreciation of relational aspects (eg, belonging and acceptance), and appreciation for useful resources and advice | — | — |
| Smedly et al [26] | Social support development | — | Frequency of types of posts aligned to different categories derived from the Social Support Behavior Code | Emotional supportive posts: 73.8%  Empathy: 66.1%  Physical affection: 13.6%  Encouragement: 13.1%  Relationship: 5.4%  Sympathy: 2.7%  Informational support: 34.8%  Advice: 22.2%  Situation appraisal: 12.2%  Teaching: 4.5%  Referral: 2.7%  Esteem support: 31.2%  Validation: 22.6%  Compliment: 11.3%  Network supports: 7.2%  Companions: 5.4%  Presence: 1.4%  Access: 0.5%  Tangible aid: 2.3%  Direct task: 1.4%  Indirect task: 0.5%  Willingness: 0.5% |
| van Uden-Kraan et al [17] | Patient empowerment | Better informed about disease and assistive devices, more confident in the relationship with their physician, more confident about treatment decision making, acceptance of the disease and coping strategies, dealing with the social environment, increased optimism and control, enhanced self-esteem, social well-being, and collective action | — | — |
| van Uden-Kraan et al [36] | Patient empowerment | — | 5-point scale (1=completely disagree and 5=completely agree) | Posters, mean (SD) versus lurkers, mean (SD; *P* value)  Being better informed: 3.7 (0.8) versus 3.6 (0.7; *P*=.03)  Enhanced social well-being: 3.4 (1.0) versus 2.8 (0.8; *P*<.001)  Feeling more confident with their physician: 3.4 (0.7) versus 3.3 (0.6; *P*=.6)  Improved acceptance of disease: 3.3 (0.9) versus 3.1 (0.9; *P*=.15)  Feeling more confident about treatment: 3.2 (0.8) versus 3.1 (0.8; *P*=.13)  Enhanced self-esteem: 3.2 (0.9) versus 3.0 (0.8; *P*=.05)  Increased optimism and control: 3.2 (0.6) versus 3.1 (0.6; *P*=0.16) |
| van Uden-Kraan et al [18] | Patient empowerment | — | 5-point scale (1=completely disagree and 5=completely agree) | Arthritis, mean (SD) and fibromyalgia, mean (SD)  Being better informed: 3.5 (0.7) and 3.8 (0.7)  Enhanced social well-being: 3.2 (0.8) and 3.4 (0.9)  More confident with physician: (0.6) and 3.4 (0.7)  Improved illness acceptance: 3.2 (0.7) and 3.4 (0.8)  More confident about treatments: 3.1 (0.8) and 3.4 (0.7)  Increased control: 3.0 (0.5) and 3.3 (0.7)  Enhanced self-esteem: 2.9 (0.9) and 3.3 (0.9) |
| Willis [21] | Health literacy development | Members construct personal disease management programs based on shared personal experience and negotiation of disability; members relate disease activity, including disease diagnosis, symptom flares, and mood, as a means of problem solving; and members share personal experiences (ie, modeling and observe others’ success and failure) | — | — |
| Willis [20] | Self-management processes | Sharing disease experiences from disease veterans to newly diagnosed members; using peer network to ask for encouragement, reassurance, advice, and positive reinforcement when experiencing disease symptoms; and members provided advice based on their own experience, which encouraged and discouraged others from engaging in particular behaviors | — | — |
| Willis and Royne [22] | Self-management processes | — | Number of self-management behaviors, type of self-management behaviors, and number of perceived benefits | Mean index (SD)  WebMD: 2.94 (1.65)  Creaky Joints: 1.88 (1.51)  About.com: 1.33 (1.22)  Arthritis Foundation: 1.11 (1.13)  Percentage of posts (across all online support groups)  Information: n=545 (28.8%)  Drug management: n=575 (29.3%)  Symptom management: n=444 (22.7%)  Psychological consequences: n=136 (6.9%)  Lifestyle factors: n=183 (9.3%)  Social support: n=522 (16.6%)  Communication: n=327 (16.7%)  (3) Most frequently reported perceived benefits (n=851)  Increased mobility, flexibility, less pain, more energy, feelings of happiness, and positive prognosis |
